# The impact of PAP therapy first impression on short-term treatment adherence

**DOI:** 10.1007/s11325-025-03320-4

**Published:** 2025-04-09

**Authors:** Monica Amendolara, Valentina Di Lecce, Carla Santomasi, Vitaliano Nicola Quaranta, Andrea Portacci, Ilaria Dei Lazzaretti, Laura Anna Sara Cuccaro, Massimo Casparrini, Sebastiano Spierto, Vito Picerno, Cristina De Robertis, Sara Quaranta, Silvano Dragonieri, Giovanna Elisiana Carpagnano

**Affiliations:** Institute of Respiratory Disease, Department of Translational Biomedicine and Neuroscience, University “Aldo Moro”, 70124 Bari, Italy

**Keywords:** CPAP, Adherence, Questionnaire, Interface, Pressure, Tolerance

## Abstract

**Purpose:**

Positive airway pressure (PAP) treatment for obstructive sleep apnea syndrome (OSAS) is often burdened by lower adherence rates. Patients’ perception and acceptance of PAP therapy play a crucial role in achieving acceptable adherence.

**Method:**

We conducted a prospective cohort study to assess patients’ initial impressions of PAP therapy using a six-item questionnaire. The questionnaire evaluated CPAP tolerance, interface comfort, titration pressure, likelihood of CPAP use, expected health benefits, and overall attitude toward PAP therapy. Patients underwent a baseline awake PAP therapy trial (T0) followed by titration with an automatic device (APAP). After one month of titration, a fixed CPAP value was set (T1). Follow-ups occurred at 1 (T2), 3 (T3), and 6 (T4) months after the start of treatment. Adherence to PAP therapy was considered sufficient if the mean device usage was ≥ 4 h/night at T4.

**Results:**

After six months, 77% of the enrolled patients achieved high PAP treatment adherence. Questionnaire scores generally improved from T0 to T4, particularly in CPAP tolerance, likelihood of treatment adherence, expected health benefits, and overall judgment of PAP therapy. Time-to-event analysis revealed that higher baseline scores in titration pressure comfort, likelihood of CPAP adherence, and overall judgment of PAP therapy were significantly associated with higher adherence likelihood.

**Conclusion:**

Patients’ first judgement on PAP therapy could significantly influence short-term adherence. Early identification and management of patients’ complaints and discomforts could improve adherence rates and PAP perception over time.

**Supplementary Information:**

The online version contains supplementary material available at 10.1007/s11325-025-03320-4.

## Introduction

Obstructive sleep apnea syndrome (OSAS) is characterized by recurrent episodes of partial or complete collapse of the pharyngeal airway during sleep, followed by periods of normal breathing resumption [1]. Repeated episodes of upper airway collapse lead to intermittent hypoxia and/or fragmented sleep, causing daytime and nighttime symptoms which can severely affect patients’ quality of life [2]. Currently, the gold standard for OSAS treatment is continuous positive airway pressure (CPAP) therapy [3]. Therapeutic pressure titration is frequently ensured by performing a home titration with automatic CPAP (APAP) instead of using in-lab strategies, which would require more time away from home for the patients and higher costs for the healthcare system [3]. Proper APAP titration involves several steps like selecting the most suitable interface, educating the patient about the use of the device and all the associated consumable materials and addressing early possible discomforts related to CPAP use [4]. However, CPAP treatment is frequently burdened by low adherence rates [5]. Factors associated with CPAP therapy adherence include sociodemographic characteristics, disease severity, psychosocial factors and therapy side effects [6]. From this standpoint, up to 30% of patients refuse CPAP therapy without a trial, while 25% of those starting the treatment spontaneously cease CPAP use within the first year [6]. Moreover, the use of CPAP therapy in the first month of therapy can foster long-term adherence, so early supportive interventions could improve patients’ adherence and outcomes [5, 7, 8].

Despite this evidence, there is few data in literature reporting the impact of patients’ perception of the first CPAP trial on future treatment adherence. The aim of this study is to clarify whether the patient’s first impression on CPAP titration could predict short-term treatment adherence.

## Materials and methods

### Study design

We conducted a prospective, single-center, observational study from January to November 2024, enrolling 140 consecutive patients with obstructive sleep apnea syndrome (OSAS) from the Sleep Disorders Clinic at Bari University Hospital.

Two dedicated pulmonologists with experience in sleep disorders (M.A. and A.P.) were in charge of the baseline and the follow up evaluations for the whole duration of the study.

OSAS was diagnosed using home sleep apnea testing (HSAT) in accordance with the recommendations of the American Association of Sleep Medicine (AASM) [9]. Patients with chronic obstructive pulmonary disease (COPD), obesity hypoventilation syndrome (OHS), or neuromuscular diseases were excluded. Additionally, patients who declined participation in the study, patients without baseline and/or follow-up questionnaires or without CPAP follow up use data were excluded from the analysis.

The study was approved by the institutional ethics committee of our center (ethics committee number: 6750) and was conducted in accordance with the Declaration of Helsinki (1975) and Good Clinical Practice standards. Written informed consent was obtained from all patients prior to enrollment.

### CPAP titration strategy

Following the diagnosis of OSAS, a baseline visit (T0) was conducted, during which patients underwent their first APAP trial. This visit included structured educational training that covered the therapeutic benefits of CPAP, potential risks associated with non-adherence, proper use of device components and awareness of possible side effects.

The most comfortable interface was selected in collaboration with the patient, who practiced mask positioning through multiple trials to achieve an optimal fit. This process aimed to help them confidently manage mask placement and minimize excessive leaks at home.

For the first awake trial, APAP was set in the range 5–8 cmH20 and patients were asked to use the device for 10 min.

A follow-up visit was scheduled 1 month after the initiation of regular APAP use. During this second evaluation (T1), data stored in the device memory was retrieved and analyzed using specialized software (ResScan, ResMed Corp. San Diego, CA, USA; PrismaTS, Löwenstein Medical SE & Co. KG, Germany). At T1, we addressed any issues or discomforts reported by patients (see Supplemental Material), verifying APAP adherence with the device’s built-in time counter. Then, according to 95th percentile (P95) of airway pressure values and after visual analysis of APAP data, patients were switched to a fixed CPAP pressure or maintained APAP mode according to their comorbidities, tolerance, response to symptoms and clinical judgement [3]. Adjustments to CPAP humidity levels, ramp time or other settings were made on a case-by-case basis.

The subsequent follow-up assessments were scheduled at one month (T2), three months (T3), and six months (T4) from T1. At each follow-up visit, clinicians assessed residual symptoms, discomforts, and any other issues reported by patients.

High PAP adherence was defined if the mean device usage from baseline to the last follow-up visit was ≥ 4 h/night.

### Patients’ perspective on PAP therapy

All enrolled patients were asked to complete a questionnaire to evaluate their perception of PAP therapy at every follow-up visit. The questionnaire, originally proposed by Balachandran et al. in a 2013 pilot study [10], included six questions addressing different aspects of CPAP treatment (Q1 = CPAP tolerance, Q2 = interface comfort, Q3 = titration pressure, Q4 = likelihood of CPAP use, Q5 = expected health benefits, Q6 = overall attitude towards CPAP therapy). Each question was rated on a 1–10 scale. A score of ≥ 6 on each item was defined as a "high" score, while a total ≥ 36, calculated as the sum of individual item scores, was considered sufficient. At T0, we considered for Q3 the pressure used for the first CPAP awake trial. For Q5, patients were briefly instructed on which daytime and nighttime symptoms could improve with CPAP regular use (see eTable 1). Patients were then invited to subjectively express Q5 score at each follow up visit.

### Enrollment process

**Fig. 1:**
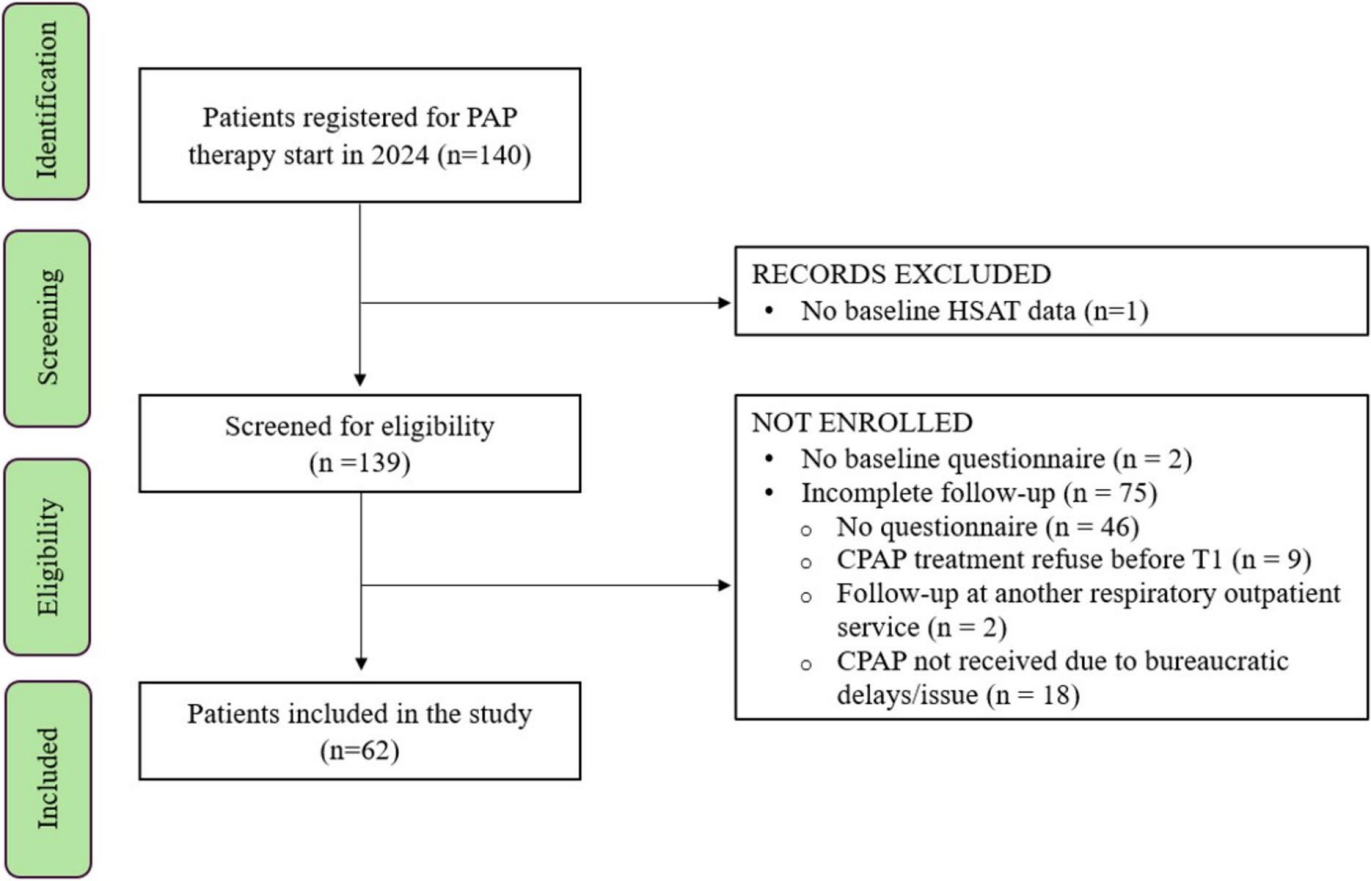
Flow chart showing the enrollment process for the overall population

Among patients registered for PAP titration, one was excluded due to incomplete HSAT data. The remaining 139 patients were assessed for exclusion criteria (see Fig. 1). Two patients did not complete their questionnaire at T0, while 75 dropped out before completing their follow-up. Among them, 46 missed their questionnaire at least once between T1 and T4, nine refused CPAP treatment before the T1 visit, and two chose to continue follow-up at another respiratory outpatient service. Additionally, 18 patients did not receive a CPAP device due to bureaucratic delays or issues. For patients with missing questionnaires scores, we were able to retrieve data on CPAP adherence from 39 of them, while 7 patients did not attend their scheduled follow-up and were not included in our telemonitoring system.

### Statistical analysis

After verifying the sample distribution with the Shapiro–Wilk test, variables with a normal distribution were presented as means ± standard deviations (SD), while those with a non-normal distribution were reported as medians and interquartile ranges (IQR). For comparisons between continuous variables, we used the Kruskal–Wallis test and the Wilcoxon test. Categorical variables were expressed as percentages and analysed using either Fisher’s exact test or the Chi-square test.

To evaluate the linear correlation between individual questionnaire items at T0 and adherence to PAP therapy at T4, we performed Spearman’s correlation test.

Finally, Kaplan–Meier time-to-event analysis with log-rank tests was used to estimate the probability of adherence to PAP therapy based on T0 high scores for the various items in the studied questionnaire.

All statistical analyses were performed using SPSS 26.0 (SPSS Inc, Chicago, Illinois) and R software (version 4.0.2, R Foundation), considering a *p*-value < 0.05 as statistically significant.

## Results

We enrolled a total of 62 consecutive patients for the final analysis (see Fig. 1). Baseline demographic and clinical characteristics are shown in Table 1. Most of the enrolled patients were male (66% vs 34%) with a mean age of approximately 63.5 years (IQR 56–69). From a clinical standpoint, patients were frequently overweight or obese, while only 22.6% reported significant excessive daytime sleepiness at baseline, defined by an Epworth Sleepiness Scale (ESS) score ≥ 10. After performing diagnostic HSAT, more than half of the enrolled patients were diagnosed with severe OSAS (58%). Nevertheless, patients with mild OSAS (19.4%) were still deemed eligible for PAP therapy due to the presence of significant comorbidities or symptoms.

**Table 1:** Baseline anthropometric and clinical features in the enrolled population

	**Enrolled population**
Patients (n)	62
Age (years)	63.5 [56–69]
Sex (M/F, %)	66/34
BMI (kg/m^2^)	29 [27–35]
Neck circumference (cm)	41 [38–44]
ESS	5 [3–10]
ESS ≥ 10 (%, n)	22.6 (14)
STOP-BANG questionnaire	5 [4–6]
Home Sleep Apnea Testing (HSAT)	
AHI (events/h) ODI (events/h) TST90 (%) Mean SpO2 (%) Lowest SpO2 (%)	35.1 [18.6–50.6] 35.1 [18.7–45.4] 8.1 [2.2–13.8] 92.5 [92–94] 80 [73–84]
OSAS severity (%, n)	
Mild Moderate Severe	19.4 (12) 22.6 (14) 58 (36)

*AHI* apnea–hypopnea index, *BMI* body mass index, *ESS* Epworth sleepiness scale, *ODI* oxygen desaturation index, *OSAS* obstructive sleep apnea syndrome, STOP-BANG snoring tiredness observed-apneas pressure BMI age neck circumference gender, TST90 total sleep time with SpO2 < 90%

After 6 months of treatment, 77.4% of the enrolled patients showed good CPAP adherence, with a median use of 5 h/night (see Table 2). Median residual AHI registered by PAP devices was 1.2 events/hour (IQR 0.6–2.2 events/hour), while the 95th percentile of unintentional leaks was 18.6 (IQR 11.1–28.6). At T4, 38.7% of the patients reported at least one reason for discomfort related to PAP therapy, with dry nose or mouth being the most frequently reported (22.6%).

**Table 2:** CPAP titration data after 6 months of treatment

CPAP use (h)	5 (4–6)
High PAP adherence (n, %)	77.4 (48)
CPAP titration pressure (cmH20)	10 [8–11]
Registered residual AHI (events/h)	1.2 [0.6–2.2]
Unintentional leaks (95th percentile)	18.6 [11.1–28.6]
Reported discomforts (%, n)	
Dry nose/mouth Interface Unintentional leaks Aerophagy Claustrophobia Nasal congestion High titration pressure Low titration pressure Noise Insomnia Bed partner discomfort	22.6 (14) 1.6 (1) 6.5 (4) 1.6 (1) 0 1.6 (1) 1.6 (1) 0 4.8 (3) 1.6 (1) 3.2 (2)

*AHI* apnea–hypopnea index, *CPAP* continuous positive airway pressure

**Fig. 2:**
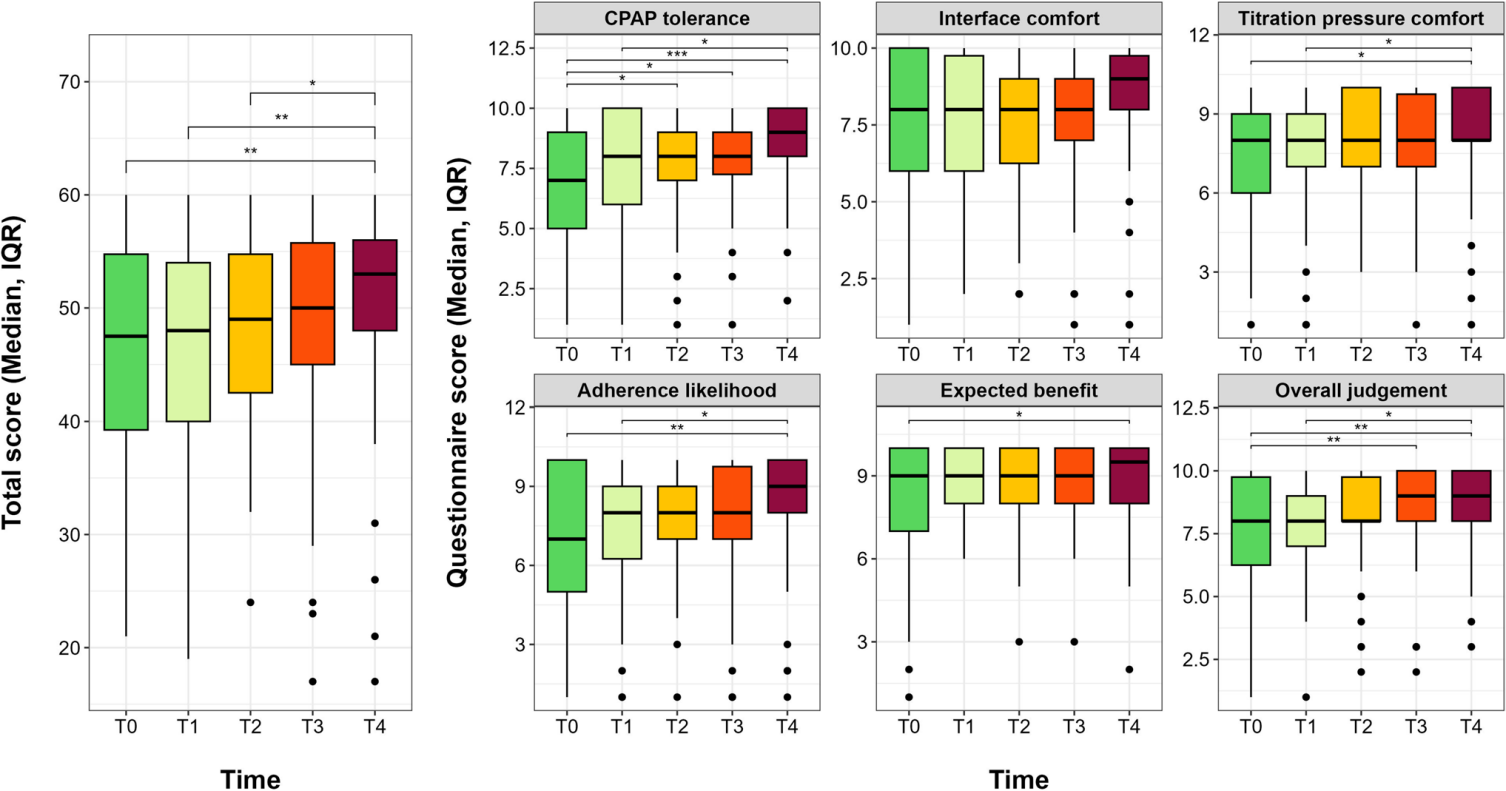
Median differences in questionnaires’ items and total score from T0 to T4. * *P* < 0.05; ** *P* < 0.01; **** *P* < 0.0001

**Fig. 3:**
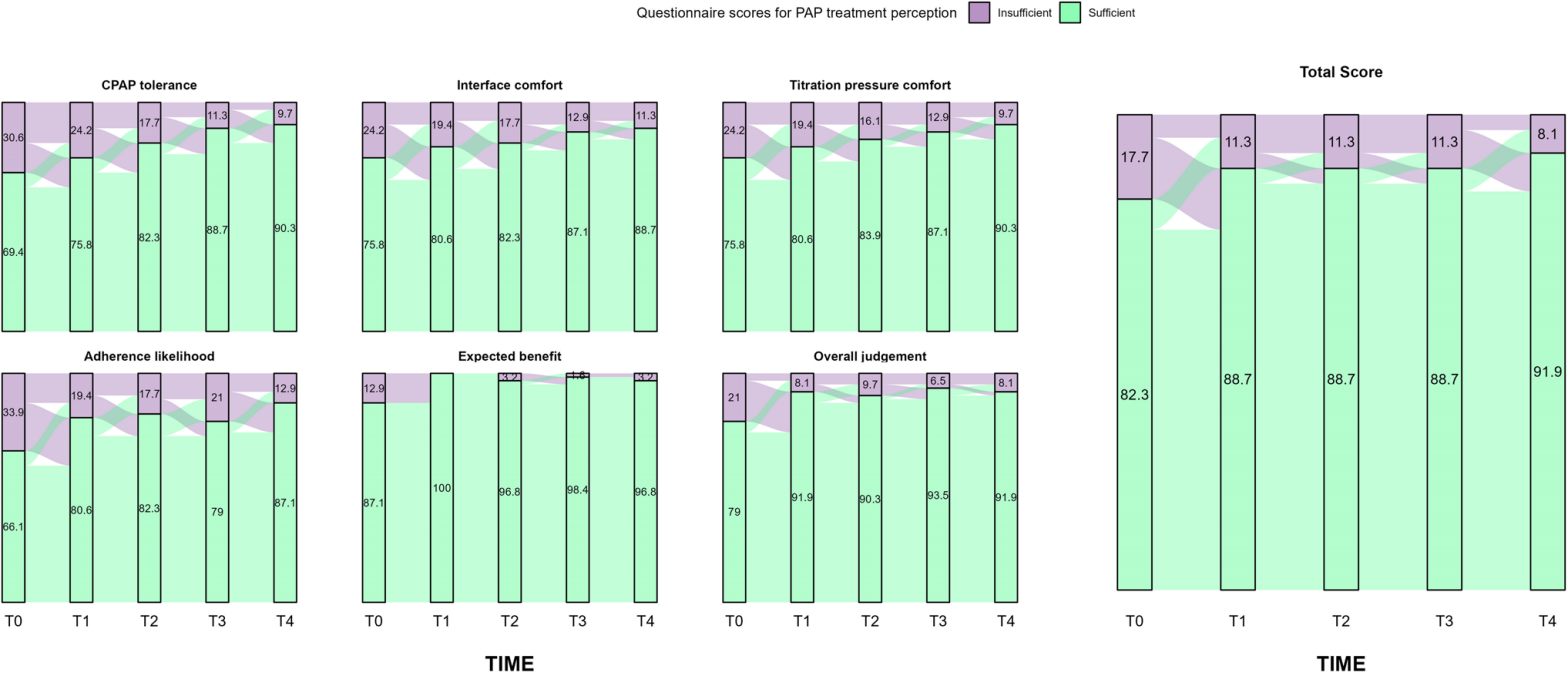
Sankey plot for the change in questionnaires’ scores (≥ 6 points vs < 6 points) from T0 to T4

Regarding the questionnaire on PAP treatment perception, all the items’ scores increased from T0 to T4, considering both their median values and the percentage of high responses at each timepoint. However, patients’ perspectives on CPAP tolerance (*p* = 0.006), likelihood of CPAP adherence (*p* = 0.048), and overall judgment on PAP therapy (*p* = 0.01) significantly improved along the follow-up (see eTable 2, Fig. 2). Similarly, a statistically significant shift towards a higher score in CPAP tolerance (*p* = 0.01) and expected health benefit using PAP therapy (*p* = 0.006) was reported from T0 to T4 (see eTable 3, Fig. 3).

To outline the association between the questionnaire’s items and adherence to PAP therapy, we performed Spearman’s correlation analysis (see eTable 4). Our results revealed a weak positive relationship between the registered hours of CPAP use and the overall judgment on PAP treatment (ρ = 0.31, *p* = 0.01) as well as the total questionnaire score (ρ = 0.28, *p* = 0.03).

**Fig. 4:**
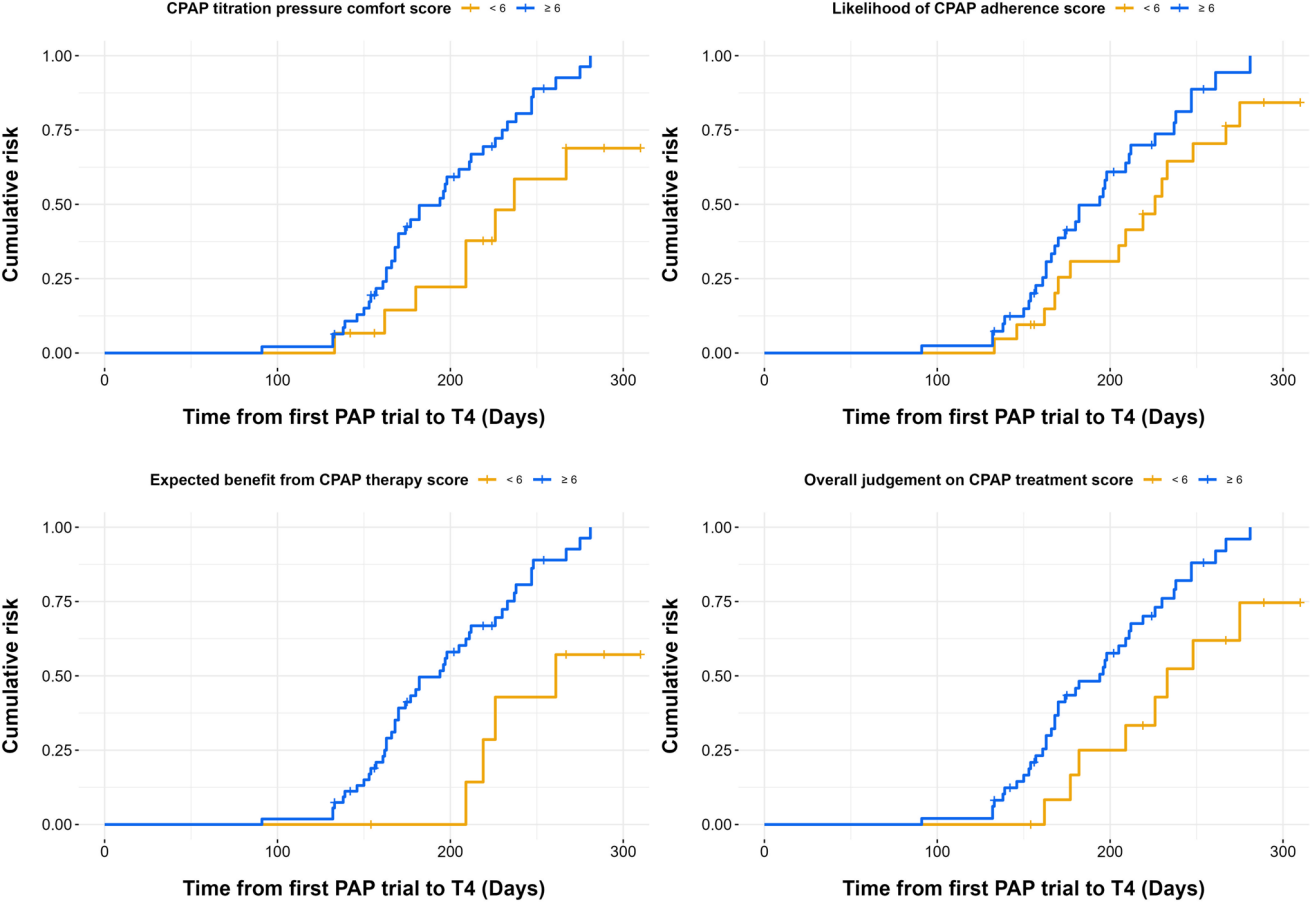
Kaplan–Meier curve for good positive airway pressure (PAP) adherence based on T0 ratings of titration pressure judgement, likelihood of PAP adherence, expected benefit from PAP therapy and overall judgement on PAP treatment

Finally, in time-to-event analysis, higher baseline scores in titration pressure comfort (*p* = 0.01), likelihood of CPAP adherence (*p* = 0.04), expected health benefit (*p* = 0.006), and overall judgment of PAP treatment (*p* = 0.008) were significantly associated with a higher likelihood of good adherence to CPAP therapy (see Table 3, Fig. 4). To assess the robustness of our findings, we conducted a sensitivity analysis with Kaplan–Meier curves, incorporating patients who had dropped out due to the lack of follow-up questionnaires but had retrievable data on CPAP adherence (39 patients). Baseline scores for titration pressure comfort (χ^2^ = 6.8, *p* = 0.009), expected health benefit (χ^2^ = 4.5, *p* = 0.03), and overall judgment of PAP treatment (χ^2^ = 5.2, *p* = 0.02) remained significantly associated with treatment adherence. However, the likelihood of CPAP adherence exhibited only a positive trend toward significance (χ^2^ = 2.2, *p* = 0.14).

**Table 3:** Kaplan–Meier analysis for CPAP adherence and baseline questionnaires responses

Baseline questionnaire	χ2	*P* value
CPAP tolerance Interface comfort Titration pressure comfort Likelihood of CPAP adherence Expected health benefit Overall judgement Total	0.05 0.94 6.3 4.1 7.6 7.1 0.96	0.82 0.33 **0.01** **0.04** **0.006** **0.008** 0.33

*CPAP* continuous positive airway pressure

## Discussion

In this study, we investigated whether a patient’s first impression of CPAP therapy could influence short-term adherence to treatment. To assess this, we employed a 6-item questionnaire addressing key aspects of PAP treatment, including titration pressure comfort, device and interface tolerance, likelihood of therapy adherence, expected health benefits, and overall judgment of CPAP therapy. At baseline, high scores were not evenly distributed among the questionnaire items. Notably, nearly one-third of the patients reported lower scores for CPAP tolerance and the likelihood of adherence, while most patients expected health benefits and expressed a positive overall judgment of PAP treatment. However, all item scores improved during follow-up evaluations, suggesting that timely interventions and close follow-ups could enhance patients’ perceptions of CPAP therapy. Since patients may face various challenges during the initial months of CPAP adaptation, it is critical to ensure consistent support from dedicated, experienced healthcare professionals (HCPs). From this perspective, standardized follow-up programs with early interventions to resolve CPAP-related issues can improve adherence and reduce voluntary treatment abandonment [11].

CPAP adherence rates are estimated to be around 50%, with nearly one-third of patients refusing treatment from the onset [4, 12]. In our enrolled cohort, 77% of patients achieved high CPAP adherence and none voluntarily discontinued CPAP therapy within six months. However, among the patients excluded due to incomplete follow-up, adherence was slightly lower (26 out of 39 patients, 66.7%). Additionally, 9 patients refused CPAP treatment before the first follow-up visit (T1), resulting in an overall adherence rate of 54.2%. As previously highlighted in literature [13, 14], factors such as the type of healthcare professionals delivering the treatment, their training and expertise in motivational strategies may influence the effectiveness of the intervention. For baseline and follow-up evaluations we ensured continuous support by involving two dedicated sleep disorder pulmonologists (M.A. and A.P.). Both in-person and remote visits were utilized to maintain close contact with patients and address their needs promptly. Although this strategy could influence treatment adherence, some patients still refused CPAP use in favours of other therapeutic strategies. Nevertheless, clinicians should always take patients’ preferences into account and explore alternative therapeutic strategies when CPAP therapy is declined.

When comparing baseline questionnaire scores with CPAP adherence at T4, we found that overall judgment of PAP therapy and the total questionnaire score exhibited a weak linear correlation with hours of CPAP use. These findings highlight the multifaceted nature of patients’ first impressions of CPAP therapy, suggesting that several aspects are valued differently by patients and may non-linearly influence both short and long-term adherence. Nevertheless, as demonstrated in our time-to-event analysis, reporting higher scores for items Q3, Q5, and Q6—covering titration pressure comfort, likelihood of CPAP use, and overall attitude towards CPAP therapy—was associated with better CPAP adherence.

Previous studies have indicated that the initial steps in CPAP adaptation may predict long-term adherence, since even the first night of CPAP trial can foreshadow future device use [5, 15, 16]. However, our study expands on this by demonstrating that even a brief PAP trial in awake patients can effectively predict short-term adherence based on patients’ perceptions of CPAP therapy. Moreover, the presence of two experienced clinicians for patient education and follow-up ensured a standardized approach, which may have facilitated HCP-patient trust and possibly supported better treatment adherence. Our results differ slightly from those of Balachandran et al., who identified a significant correlation between CPAP tolerance (Q1) and CPAP adherence [10]. While their analysis relied on Pearson correlation, we employed Kaplan–Meier curves with log-rank tests to assess consistency over time. Similarly, a study conducted by Van Zeller et al. followed 88 male patients with moderate-to-severe OSAS for 5 years after initiating APAP therapy. Their findings showed a 77% adherence rate at 5 years, with a higher risk of poor long-term adherence observed among patients with low CPAP usage during the first 12 h and within the first 6 months of therapy [17].

From this perspective, several studies have investigated potential predictors of poor adherence to CPAP therapy. In a study conducted by Borel et al. on a population of 2311 patients with OSAS, risk factors for non-adherence were grouped into three domains: general patient characteristics, disease severity, and technical aspects of treatment [18]. Within the latter domain, factors significantly associated with lower adherence included the type of interface, titration pressure and certain side effects such as nasal congestion, dry mouth, and perceived psychological discomfort [18]. In our study, interface comfort was not significantly associated with lower adherence, likely due to the collaborative selection of the interface during the initial evaluation. This process involved tailoring choices to individual patient characteristics and preferences. Moreover, in cases of reported discomfort during follow-up, prompt interventions by dedicated staff minimized time spent using an uncomfortable interface. However, trials using different CPAP masks are often prohibitive due to high costs and current insurance policies. Expanding insurance coverage to allow the use of various interfaces in CPAP trials could enhance adherence and improve long-term outcomes.

The importance of CPAP pressure comfort further highlights the need for well-trained and dedicated HCPs in sleep centres. Titration pressure selection, often based on automated APAP reports, must not overlook essential steps such as downloading device memory data, analyzing flow curves, detecting unintentional leaks, verifying the resolution of respiratory events during sleep, and, most importantly, considering patient preferences. Equally important is patient education about the positive health effects of rigorous CPAP therapy. Patients should be informed about the therapy’s long-term benefits in managing comorbidities, as well as its short-term impact on improving individual symptoms [19]. Finally, the overall judgment of therapy reflects the combined evaluation of various aspects of treatment, including ease of use, integration into daily routines, bureaucratic challenges (e.g., initial supply and license renewal), side effects, sources of discomfort, and perceived benefits. Preparing patients for these challenges before starting therapy enables early and effective management as treatment progresses.

Our study has some limitations, including its single centre nature, the short follow up time and the relatively small sample size and. Moreover, while the sensitivity analysis showed consistent results when including patients with adherence data but missing follow-up questionnaires, we cannot exclude the possibility that patients without adherence data or those with delayed CPAP setup may have had different experiences or outcomes.

Another important aspect of our study was the decision to assign two dedicated pulmonologists to provide patient support, which may have positively influenced patients’ first impressions of CPAP therapy. However, relying on two dedicated specialists for this task could present feasibility challenges, particularly in terms of associated costs. However, we chose this approach to standardize the follow-up process, considering the subjective nature of the questionnaire and its potential relationship with HCP-patient interactions.

In conclusion, our study highlights the relationship between patients’ first perception of CPAP therapy and short-term treatment adherence. Patients who experience less discomfort during PAP titration and provide a positive initial assessment of therapy during their first CPAP trial are more likely to achieve good short-term adherence. Further studies are needed to determine whether early interventions could modify adherence trajectories, particularly for patients at the highest risk of treatment failure.

## Supplementary Information

Below is the link to the electronic supplementary material.

Supplementary file1 (DOCX 56 KB)

Supplementary file2 (DOCX 35 KB)

## Data Availability

The data that support the findings of this study are available from the corresponding author upon reasonable request.

## Abbreviations

APAP: Automatic positive airway pressure
CPAP: Continuous positive airway pressure
OSAS: Obstructive sleep apnea syndrome
COPD: Chronic obstructive pulmonary disease
HSAT: Home sleep apnea testing
HCPs: Healthcare professionals
IQR: Interquartile ranges
OHS: Obesity hypoventilation syndrome
SD: Standard deviations
