# Supplementary material for: The impact of PAP therapy first impression on short-term treatment adherence
Source: Sleep Breath. 2025 Apr 9;29(2):152. doi: 10.1007/s11325-025-03320-4 (PMC11978682; doi:10.1007/s11325-025-03320-4)
Supplement: Supplementary file 1 — Supplementary file1 (DOCX 56 KB) [file 11325_2025_3320_MOESM1_ESM.docx]

**Title:** The impact of PAP therapy first impression on short-term treatment adherence.

**Short title:** First impression of PAP therapy on adherence

**Monica Amendolara^1*^, Valentina Di Lecce^1*^, Carla Santomasi^1^, Vitaliano Nicola Quaranta^1^, Andrea Portacci^1#^, Ilaria Dei Lazzaretti^1^, Laura Anna Sara Cuccaro^1^, Massimo Casparrini^1^, Sebastiano Spierto^1^, Vito Picerno^1^, Cristina De Robertis^1^, Sara Quaranta^1^, Silvano Dragonieri^1^, Giovanna Elisiana Carpagnano^1^**

^1^Institute of Respiratory Disease, Department of Translational Biomedicine and neuroscience, University “Aldo Moro”, Bari, Italy

* These authors contributed equally to this manuscript

^#^ Corresponding author: Andrea Portacci

70124 Bari, Italy

Tel. +39 3355854523

Email: [a.portacci01@gmail.com](mailto:a.portacci01@gmail.com)

**SUPPLEMENTAL MATERIAL**

**Supplemental table**

**Supplemental table 1**. OSAS-related daytime and nighttime symptoms described explaining the item Q5 “expected health benefit” from the questionnaire.


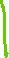


| **Daytime Symptoms**  Sleepiness  Fatigue  Headache  Insomnia  Memory Loss  Concentration disorders  Mood disorders |
| --- |
| **Nighttime symptoms**  Snoring  Reported Apneas  Choking  Nocturia  Awakenings  Sweating |

**Supplemental table 2**. PAP treatment perception questionnaire at baseline and over follow up


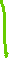


|  | Baseline | APAP titration | 1 month CPAP therapy | 3-month CPAP therapy | 6-month CPAP therapy | P value |
| --- | --- | --- | --- | --- | --- | --- |
| **Baseline questionnaire (Median, IQR)**   - CPAP tolerance - Interface comfort - Titration pressure comfort - Likelihood of CPAP adherence - Expected health benefit - Overall judgement - Total | 7 [5-9.2]  8 [5.7-10]  8 [5.7-9]  7 [5-10]  9 [7-10]  8 [6-10]  47.5 [39-55.2] | 8 [5.7-10]  8 [6-10]  8 [7-9]  8 [6-9]  9 [8-10]  8 [7-9.5]  48 [40-54.2] | 8 [7-9.2]  8 [6-9]  8 [7-10]  8 [6.7-9]  9 [8-10]  8 [8-10]  49 [42-55] | 8 [7-9]  8.5 [7-10]  8 [7-10]  8.5 [6.7-10]  9 [8-10]  9 [8-10]  50.5 [44.7-56] | 8 [9-10]  9 [8-10]  8 [7.7-10]  9 [8-10]  9.5 [8-10]  9 [8-10]  53 [47.7-56.2] | **0.006**  0.35  0.12  **0.048**  0.12  **0.01**  **0.01** |

**Supplemental table 3**. Frequency of sufficient results to PAP treatment perception questionnaire. For single items a score ≥ 6 points was considered as sufficient, while for the total score a sufficient result was considered ≥ 36 points


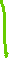


|  | Baseline | APAP titration | 1 month CPAP therapy | 3 months CPAP therapy | 6 months CPAP therapy | P value |
| --- | --- | --- | --- | --- | --- | --- |
| **Baseline questionnaire (%, n)**   - CPAP tolerance - Interface comfort - Titration pressure comfort - Likelihood of CPAP adherence - Expected health benefit - Overall judgement - Total | 69.4 (43)  75.8 (47)  75.8 (47)  66.1 (41)  87.1 (54)  79 (49)  82.3 (51) | 75.8 (47)  80.6 (50)  80.6 (50)  80.6 (50)  100 (62)  91.9 (57)  88.7 (55) | 82.3 (51)  82.3 (51)  83.9 (52)  82.3 (51)  96.8 (60)  90.3 (56)  88.7 (55) | 88.7 (55)  87.1 (54)  87.1 (54)  79 (49)  98.4 (61)  93.5 (58)  88.7 (55) | 90.3 (56)  88.7 (55)  90.3 (56)  87.1 (54)  96.8 (60)  91.9 (57)  91.9 (57) | **0.01**  0.33  0.22  0.07  **0.006**  0.1  0.6 |

**Supplemental table 4**. Correlation between PAP treatment perception questionnaire with hours of PAP use


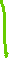


|  | Spearman ρ | P value |
| --- | --- | --- |
| **Baseline questionnaire (Median, IQR)**  CPAP tolerance  Interface comfort  Titration pressure comfort  Likelihood of CPAP adherence  Expected health benefit  Overall judgement  Total | 0.17  0.21  0.24  0.23  0.17  0.31  0.28 | 0.19  0.1  0.06  0.07  0.19  **0.01**  **0.03** |

**Supplemental table 5**. Baseline features of the overall population. Data includes the 62 enrolled patients and the 39 who did not complete their follow-up but had retrievable data on treatment adherence.


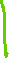


|  | **Overall population** |
| --- | --- |
| Patients (n) | 101 |
| Age (years) | 61.5 [54-68.7] |
| Sex (M/F, %) | 69.3/30.7 |
| BMI (kg/m^2^) | 30 [27-36] |
| Neck circumference (cm) | 41 [38-45] |
| ESS | 5 [3-10] |
| ESS ≥10 (%, n) | 22.8 (23) |
| STOP-BANG questionnaire | 5 [4-6] |
| **Home Sleep Apnea Testing (HSAT)**  AHI (events/h)  ODI (events/h)  TST90 (%)  Mean SpO2 (%)  Lowest SpO2 (%) | 34.8 [19.9-49.8]  34.9 [20.1-45.2]  6.4 [2.2-15.3]  92.7 [91.8-94]  80 [74-83] |
| **OSAS severity (%, n)**  Mild  Moderate  Severe | 58.5 (59)  25.7 (26)  15.8 (16) |
| **Baseline questionnaires scores (Median, IQR)**  CPAP tolerance  Interface comfort  Titration pressure comfort  Likelihood of CPAP adherence  Expected health benefit  Overall judgement  Total | 8 [7-9]  8 [7-9]  8 [7-9]  8 [6-9]  9 [8-10]  9 [7-10]  50 [42.5-55] |
